# Reply to Austin and Korem, “Compositional transformations can reasonably introduce phenotype-associated values into sparse features”

**DOI:** 10.1128/msystems.00248-25

**Published:** 2025-05-02

**Authors:** Steven L. Salzberg

**Affiliations:** 1 Center for Computational Biology, Johns Hopkins University, Baltimore, Maryland, USA; 2 Department of Biomedical Engineering, Johns Hopkins University, Baltimore, Maryland, USA; 3 Department of Computer Science, Johns Hopkins University, Baltimore, Maryland, USA; 4 Department of Biostatistics, Johns Hopkins University, Baltimore, Maryland, USA; Medizinische Universitat Graz, Graz, Styria, Austria

## REPLY

Austin and Korem start their paper with the claim that “It was recently argued that an analysis of tumor-associated microbiome data is invalid because features that were originally very sparse (genera with mostly zero read counts) became associated with the phenotype following batch correction.” This is a misstatement of the claims in our paper. We did indeed argue that an earlier analysis was invalid, but we laid out very clearly that the earlier analysis made two major errors. Our paper describes these errors in great detail, but nowhere did we claim that the previous paper was invalid for the reason they state. Thus, they have created a straw man argument, which their paper attempts to refute in mostly theoretical terms. We have no objections to their theoretical arguments, but nothing in the paper undermines or contradicts the results of our paper. They also mention a few examples of microbes that they think might indeed be associated with cancer, such as *Thiorhodospira* in kidney cancer, one of the cases we debunked in our paper. If they truly wish to claim that *Thiorhodospira*—an extremophile bacterium that only exists in a Siberian soda lake, and nowhere else in the world—is “ highly associated” with kidney cancer, as their paper claims, we challenge them to replicate it. We stand by all of our original findings.

